# Non-fermenter Gram-negative bacilli at a tertiary hospital, South Africa

**DOI:** 10.4102/sajid.v38i1.538

**Published:** 2023-11-20

**Authors:** Sinenhlanhla Ndzabandzaba, Lesego Mothibi, Nina von Knorring

**Affiliations:** 1 Department of Clinical Microbiology and Infectious Diseases, Faculty of Health Sciences, University of the Witwatersrand, Johannesburg, South Africa; 2 Department of Microbiology Laboratory, Chris Hani Baragwanath Academic Hospital, National Health Laboratory Services, Johannesburg, South Africa

**Keywords:** prevalence, distribution, antibiogram, antimicrobial resistance, antimicrobial stewardship, infection prevention and control, non-fermenting Gram-negative bacilli

## Abstract

**Background:**

Non-fermenting Gram-negative bacilli (NFGNB) are a significant cause of healthcare-associated infections and are often implicated in nosocomial outbreaks. Non- fermenting Gram-negative bacilli tend to have variable susceptibility patterns that make the choice of empiric therapy difficult and thus treatment must be based on *in vitro* susceptibility testing of each antimicrobial agent.

**Objectives:**

To describe the epidemiology of the NFGNB isolated from adult patients at Chris Hani Baragwanath Hospital (CHBAH) and to assess their antimicrobial susceptibility patterns in order to guide empiric therapy and inform infection prevention and control practices.

**Method:**

Organisms isolated from sterile sites of adult in-patients between 01 January 2016 to 31 December 2018 were retrospectively analysed.

**Results:**

A total of 2005 NFGNB isolated. Blood cultures were the most common specimen type (91.4%). *Acinetobacter* species were the most commonly isolated organisms (65.1%), followed by *Pseudomonas* species (26.5%). The majority of NFGNB were isolated from patients in surgical wards (38.9%) followed by medical wards (35.2%). Most (60%) of the *Acinetobacter* species were extremely drug resistant. *Pseudomonas* species were more susceptible than the *Acinetobacter* species with an overall susceptibility rate of 86% for *Pseudomonas* species.

**Conclusion:**

The rates of antimicrobial resistance demonstrated among *Acinetobacter* and *Pseudomonas* species were high, which illustrates the threat of antimicrobial resistance also seen worldwide. An emergence of NFGNB with intrinsic multidrug resistance (*Stenotrophomonas maltophilia* and *Burkholderia cepacia*) was noted. We suggest empiric therapy with a carbapenem sparing regimen of piperacillin-tazobactam in combination with amikacin and that empiric therapy be reviewed annually when cumulative antibiograms are done.

**Contribution:**

Understanding of the distribution and antimicrobial susceptibility patterns of NFGNB at CHBAH.

## Introduction

The increasing emergence of multidrug-resistant organisms is a public health threat that is recognised worldwide.^1^ The World Health Organization (WHO) has published the Priority Pathogens List as part of its efforts to address the increase in the global resistance to antimicrobial agents. In the list, the threat of Gram-negative bacilli (GNB) that are resistant to multiple antibiotics is emphasised. Two non-fermenting Gram-negative bacilli (NFGNB), namely *Acinetobacter baumannii* (carbapenem resistant) and *Pseudomonas aeruginosa* (carbapenem resistant) are among the organisms in this list.^2^

Non-fermenting Gram-negative bacilli often colonise the hospital environment,^3^ hospitalised patients and the hands of healthcare workers and pose a challenge as they are resistant to a variety of disinfectants commonly used in the hospital environment.^4,5,6^ Non-fermenting Gram-negative bacilli are increasingly being cultured from normally sterile sites such as cerebrospinal fluid (CSF), blood, tissue, pus, fluid and catheter tips.^7^ Non-fermenting Gram-negative bacilli have recently been recognised as an important cause of healthcare-associated infections (HAIs) and are known to cause infections such as bacteraemia, meningitis, lower respiratory and urinary tract infections as well as wound infections^8,9^ and may be implicated in outbreaks.^10,11,12^ The role of NFGNB in causing disease is well described especially in patients who are or have been recently hospitalised.^9^ Other risk factors for infections due to NFGNB include patients that are immunocompromised (for example, oncology patients, organ transplant patients), cystic fibrosis patients, patients who have sustained trauma, mechanically ventilated patients and patients with urinary catheters.^13^

Non-fermenting Gram-negative bacilli are often inherently resistant to certain classes of antimicrobial agents.^9,14,15^ They express most of the known resistance mechanisms to antimicrobials.^16,17^ Non-fermenting Gram-negative bacilli tend to have variable susceptibility patterns, and predicting these is difficult. In recent years, there has been increased resistance to the already limited number of antimicrobials that are commonly used to treat NFGNB. One of the reasons thought to be accounting for this rise is the increased use of broad-spectrum antibiotics such as carbapenems.^18^ This emergence of resistance is being seen worldwide and has resulted in decreased treatment options as well as adverse patient outcomes.^19^ Laboratory-based sentinel surveillance for *A. baumannii* bacteraemia conducted in four South African provinces during 2018 detected 1787 cases; over a third of those whose outcome was known died in hospital.^20^

Because of the unpredictability of the susceptibility patterns, the choice of empiric therapy is complicated and must be based on *in vitro* susceptibility testing of each agent. Delay in initiating effective therapy for infections by resistant organisms has been shown to significantly increase the risk of mortality.^19^ Monitoring of emerging antimicrobial resistance trends locally using an annual summary of susceptibility rates, known as a cumulative antibiogram, is therefore critical as it guides adequate clinical management, infection-control interventions and antimicrobial-resistance containment strategies.^21^ A study in this country by von Knorring et al. showed that 15% of the NFGNB analysed were extremely drug-resistant (XDR).^16^ A similar study to ours, done in India, showed rapidly emerging resistance of the NFGNB and multidrug-resistant (MDR) rate of 35.28%.^17^ Both these studies support the use of antibiograms.

The objectives of the study therefore were to describe the epidemiology (burden and distribution) of the different NFGNB isolated from adult in-patients, as well as to assess their antimicrobial susceptibility patterns. This will assist in guiding empiric therapy for nosocomial bloodstream infections, especially in the vulnerable immunocompromised patients and in informing infection prevention and control (IPC) practices.

## Methods

This was a retrospective data analysis of laboratory records from 01 January 2016 until 31 December 2018. It was conducted at the local laboratory, in the Department of Microbiology, Chris Hani Baragwanath Academic Hospital (CHBAH). Chris Hani Baragwanath Academic Hospital is a 3400-bed tertiary hospital in Soweto, Johannesburg, South Africa. The adult wards at CHBAH are divided into medical wards (including Haematology patients), psychiatric wards, surgical wards (including burns unit), obstetrics and gynaecological wards as well as the intensive care unit (ICU). The hospital has a high prevalence of patients infected with the human immunodeficiency virus (HIV).^22^ The ICU admits trauma patients, medical patients, as well as obstetrics and gynaecology patients.

Data were extracted from the National Health Laboratory Services’ (NHLS) laboratory information system (LIS) database. Organism identification, ward and specimen type of all NFGNB from clinical specimens from sterile sites (blood, tissue, fluid and pus aspirates, central venous catheter tips) in adult patients were extracted as well as the results of antimicrobial susceptibility testing (AST). Only catheter tips with significant colony forming units (> 15 CFU) were included. We excluded samples from non-sterile sites (pus swabs, respiratory samples and urine) as well as surveillance samples. Duplicate patient isolates of the same pathogen were excluded to minimise bias due to over-representation of more resistant organisms according to the Clinical and Laboratory Standards Institute (CLSI) guidelines.^23^ For the epidemiological aspect of this study, we excluded the same species identified from the same patient within 14 days for blood cultures and 30 days for other specimens according to the Centers for Disease Control and Prevention (CDC) document on HAIs.^24^ In order to compile an antibiogram, only the first isolate of a species per patient, irrespective of body site or antimicrobial profile was included as per CLSI guidance.^25^ Species isolates for which there were less than 30 samples were excluded from the analysis as these could lead to statistical inaccuracies.^25^ We also excluded those isolates with intermediate susceptibility.^25^

Identification of the NFGNB was done using manual methods such as Analytical Profile Index (API) 20E and API 20 NE (bioMérieux, Marcy l’Étoile, France) as well as the MicroScan WalkAway (Beckman Coulter, Brea, CA, United States [US]), which is an automated method.

The AST of the isolates was performed routinely using the manual Kirby-Bauer disk diffusion method or the MicroScan WalkAway. All susceptibility results were interpreted according to the CLSI breakpoints for the corresponding year.^26,27,28^

Organisms were described as XDR if non-susceptible to ≥ 1 agent in all but ≤ antimicrobial categories or as MDR if non-susceptible to ≥ 1 agent in ≥ 3 antimicrobial categories.^29^

Data were analysed using STATA 14 statistical software. Statistical significance was determined by the use of chi-squared *p-*value at a level of significance of 0.05. The data are presented in tables and proportions after removing those that did not meet the inclusion criteria.

## Results

### Burden and distribution of non-fermenting Gram-negative bacilli

A total of 2005 NFGNB were isolated between 2016 and 2018 with blood cultures by far the most common sample type overall (*n* = 1833, 91.4%) and the most common sample type for each of the main individual species (Table 1). *Acinetobacter* species were the most commonly isolated NFGNB during the study period (*n* = 1306, 65.1%) followed by *Pseudomonas* species (*n* = 534, 26.6%), *Stenotrophomonas maltophilia* (*n* = 73, 3.6%) and *Burkholderia cepacia* (*n* = 25, 1.3%) (Table 1). This distribution was seen throughout the 3 years studied (Table 2). The other less commonly isolated NFGNB were grouped and formed 3.3% (*n* = 67) of the NFGNB.

**TABLE 1:** Organism distribution and sample type from 2016 to 2018.

Organism	Blood culture		CSF		Catheter tip		Other sterile sites†		Total	
	%	*n*	%	*n*	%	*n*	%	*n*	%	*n*
*Acinetobacter* species	91.4	1194	0.3	4	2.4	31	5.9	77	65.1	1306
*Pseudomonas* species	92.1	492	0.4	2	2.4	13	5.0	27	26.6	534
*S. maltophilia*	89.0	65	0.0	0	2.7	2	8.2	6	3.6	73
*B. cepacia*	84.0	21	0.0	0	0.0	0	16.0	4	1.2	25
Others‡	91.0	61	0.0	0	4.5	3	4.5	3	3.3	67

**Total**	**91.4**	**1833**	**0.3**	**6**	**2.4**	**49**	**5.8**	**117**	**-**	**2005**

CSF, cerebrospinal fluid.

† , Fluid/pus aspirate/tissue;

‡ , *Alcaligenes species, Aeromonas hydrophila, Brevundimonas diminuta/vesicularis*, etc. See Appendix 1.

**TABLE 2:** Yearly trends in the number of non-fermenting Gram-negative bacilli isolated from 2016 to 2018.

Organism	Number of isolates							*p*
	2016		2017		2018		Total	
	*n*	%	*n*	%	*n*	%		
*Acinetobacter* species	506	70.5	398	62.1	402	62.2	1306	0.03*
*Pseudomonas* species	168	23.4	183	28.6	183	28.3	534	0.001*
*S. maltophilia*	20	2.8	28	4.4	25	3.9	73	0.77
*B. cepacia*	6	0.8	8	1.3	11	1.7	25	0.45
Others†	18	2.5	24	3.7	25	3.9	67	0.33

**Total**	**718**	**-**	**641**	**-**	**646**	**-**	**2005**	0.04*

* , Statistically significant;

† , See Appendix 1.

The burden of the *Acinetobacter* species decreased significantly from 2016 (*n* = 506, 71%) to 2018 (*n* = 402, 62%; *p* = 0.03), whereas the total number of *Pseudomonas* species showed a significant increase during the same time period (*n* = 168, 23% and *n* = 183, 28%, respectively; *p* = 0.001) (Table 2). Most NFGNB were isolated from surgical (*n* = 780, 38.9%) and medical wards (*n* = 705, 35.2%) (Table 3). The overall majority of *Acinetobacter* species were isolated from the surgical wards (*n* = 549, 42%), whereas *Pseudomonas* species were isolated slightly more often from the medical (*n* = 208, 39%) than the surgical (*n* = 11, 35.7%) wards. The majority of the *S. maltophilia* (*n* = 29, 40%) and *B. cepacia* (*n* = 9, 36%) isolates were from the medical wards although the overall numbers were small (Table 3).

**TABLE 3:** Organisms isolated per ward from 2016 to 2018.

Organism	ICU		Medical		Obstetrics and gynaecology		Surgery		Total (*n*)
	*n*	%	*n*	%	*n*	%	*n*	%	
*Acinetobacter* species	267	20.4	411	31.5	79	6.0	549	42.0	1306
*Pseudomonas* species	88	16.5	208	39.0	47	8.8	191	35.7	534
*S. maltophilia*	17	23.3	29	39.7	4	5.5	23	31.5	73
*B. cepacia*	6	24.0	9	36.0	4	16.0	6	24.0	25
Others†	4	6.0	48	71.6	4	6.0	11	16.4	67

**Total**	**382**	**19.0**	**705**	**35.2**	**138**	**6.9**	**780**	**38.9**	**2005**

ICU, intensive care unit.

† , See Appendix 1.

### Antimicrobial susceptibility pattern of the non-fermenting Gram-negative bacilli

**FIGURE 1:**
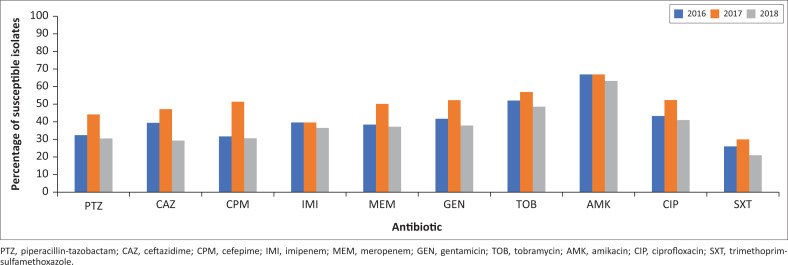
Antimicrobial susceptibilities to *Acinetobacter* species per year (2016–2018).

The antimicrobial susceptibility patterns for all isolates included in the analysis are shown in Table 4. *Acinetobacter* species showed overall low susceptibility to the antimicrobials tested exhibiting a wide range from 26% for trimethoprim – sulfamethoxazole to 71% for amikacin. There was a high incidence of XDR *Acinetobacter* species (60%) while the incidence of MDR *Acinetobacter* species was 5%. The susceptibility for piperacillin-tazobactam was similar from 2016 to 2018 (32.4% and 31%, respectively). The susceptibility rate for ceftazidime decreased by 10% from 2016 to 2018, from 39% to 29%. For cefepime, the susceptibility decreased by 20% from the year 2017 to the year 2018 (51% to 31%) (Figure 1), which was statistically significant (*p* = 0.003). Carbapenem susceptibilities for *Acinetobacter* species were 48.2% and 47.5% for surgical wards and 40.9% and 41.3% for medical wards for imipenem and meropenem, respectively. These findings are similar to the findings in the South African surveillance data of *A. baumannii* complex bacteraemia in 2017 until 2019 where the majority of the patients were paediatrics.^30^

**TABLE 4:** Antimicrobial susceptibility testing patterns of non-fermenting Gram-negative bacilli isolated from 2016 to 2018.

Organism	Antibiotic (Total number of isolates tested†; Percentage of susceptible isolates)																					
	PTZ		CAZ		CPM		IMI		MEM		GEN		TOB		AMK		CIP		SXT		Resistance phenotype (%, *n*)	
	*n*	%	*n*	%	*n*	%	*n*	%	*n*	%	*n*	%	*n*	%	*n*	%	*n*	%	*n*	%	MDR	XDR
*Acinetobacter* species	576	39.4	580	41.5	579	38.0	577	46.1	574	45.3	631	46.9	580	56.2	580	70.7	620	48.2	639	26	5§	60¶
*Pseudomonas* species	235	83.0	242	86.4	242	83.5	241	76.4	237	75.1	257	82.5	239	85.8	241	91.3	248	85.9	-	-	-	14††
*Stenotrophomonas maltophilia*	-	-	-	-	-	-	-	-	-	-	-	-	-	-	-	-	-	-	37	75.0	N/A	N/A
*Burkholderia cepacia*	-		13	69.2	-	-	-	-	12	75.0	-	-	-	-	-	-	-	-	12	58.3	N/A	N/A
Others‡	31	96.8	31	90.3	30	90.0	30	96.7	-	-	-	-	-	-	-	-	-	-	-	-	N/A	N/A

PTZ, piperacillin-tazobactam; CAZ, ceftazidime; CPM, cefepime; IMI, imipenem; MEM, meropenem; GEN, gentamicin; TOB, tobramycin; AMK, amikacin; CIP, ciprofloxacin; SXT, trimethoprim-sulfamethoxazole; MDR, multidrug resistant; XDR, extensively drug resistant.

† , Total number of isolates may differ as some isolates had missing susceptibility to some antimicrobial agents;

‡ , see Appendix 1;

§ , *n* = 28;

¶ , *n* = 346;

†† , *n* = 34.

**FIGURE 2:**
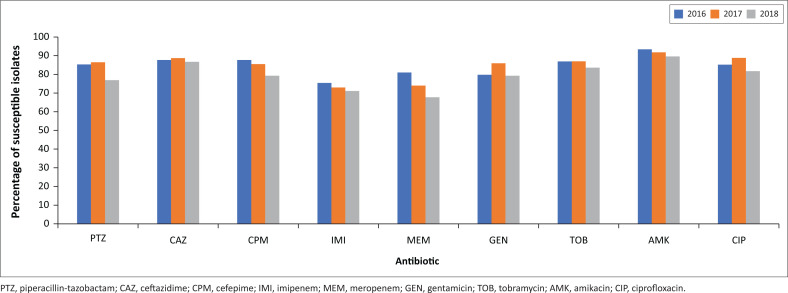
Antimicrobial susceptibilities to *Pseudomonas* species per year (2016–2018).

*Pseudomonas* species were generally more susceptible to the antimicrobials tested than were *Acinetobacter* species. The susceptibility to the two commonly used antipseudomonal agents, piperacillin-tazobactam and ceftazidime was 83% and 86%, respectively. For the antipseudomonal carbapenems, however, susceptibility was reduced (76% for imipenem and 75% for meropenem) compared to the other antibiotics tested. There was a decline in the susceptibility towards meropenem noted between 2016 (81%) and 2018 (68%), and this was statistically significant (*p* = 0.012). The highest susceptibility, as for the *Acinetobacter* species, was demonstrated for amikacin (91%) (Figure 2). The *Pseudomonas* species had susceptibilities of 80.5% and 78.9% for surgical wards, 74.9% and 72.6% medical wards for imipenem and meropenem, respectively. Neither of these were statistically significant.

*S. maltophilia* isolates showed moderate susceptibility to trimethoprim-sulfamethoxazole (TMP-SMX; 75%). Trimethoprim-sulfamethoxazole is the principal agent used for the treatment of clinically significant *S. maltophilia* infections. There was an increase in the numbers of *B. cepacia* from 2016 to 2018, but this was not statistically significant (*p* = 0.45) (Table 2).

The main agents used for the treatment of clinically significant *B. cepacia* infections showed reduced susceptibility at 58%, 69% and 75% for TMP-SMX, ceftazidime and meropenem, respectively.

## Discussion

Multidrug-resistant NFGNB have emerged as important nosocomial organisms. During the 3 years studied, the most common NFGNB isolated at CHBAH from sterile sites in adult patients were *Acinetobacter* species followed by *Pseudomonas* species. Poor susceptibility to the commonly used antimicrobials was demonstrated, especially for the *Acinetobacter* species.

The distribution of organisms noted in this study is comparable to other studies. In South Africa, the national antimicrobial resistance surveillance conducted in 2016 reported on blood cultures from 16 sentinel hospitals in the public sector (including CHBAH); of 2318 NFGNB isolates, 71% were identified as *A. baumannii* and 29% as *P. aeruginosa*,^31^ which is similar to our results (65% and 27%, respectively).

The majority of *Acinetobacter* species in our study were found in surgical wards. Our analysis showed that among ICU patients, *Acinetobacter* species were the most commonly isolated NFGNB. Similarly, a study by Ntusi et al.^32^ in Cape Town assessed adult patients in both surgical and respiratory ICUs and showed that out of 251 patients analysed, 85% of patients were infected and 15% were colonised with *A. baumannii*, the risk factors for infection being recent surgery and insertion of an endotracheal tube.^32^ The observation in our study is, therefore, also likely to be due to patients in ICU being mechanically ventilated or having post-surgical or trauma-related wound infections.

We found that most of the *Acinetobacter* species were isolated from blood culture specimens, as were the other main NFGNB species. In a study by K. Swe Swe-Han et al. looking at the clinical and microbiological characteristics and antibiotic resistance patterns of *A. baumannii* strains in both ICU and non-ICU patients from sterile and non-sterile sites, blood cultures were also reported as the commonest site of isolation in patients with sepsis (46%).^33^ *A. baumannii*, as well as certain other NFGNB colonising patients and CHBAH environment, are not always interpreted as significant pathogens in patients, and clinical characteristics have to be taken into consideration. Even samples obtained from sterile sites may be prone to contamination from the patients’ skin or the environment.

There was a significant decline noted in the number of *Acinetobacter* species from 2016 to 2018. Hospital management, nurses and doctors focused more on IPC measures during this period. These measures included educating all staff on IPC measures, hand hygiene audits, instituting stringent contact precautions where required, environmental cleaning and screening of contacts of patients infected with MDR or XDR *Acinetobacter* species. Employing strict IPC measures has been shown to significantly reduce the incidence of *A. baumannii* infections;^34^ therefore, improving IPC measures might have contributed to this finding.

The data in the study showed poor susceptibility of *Acinetobacter* species to imipenem and meropenem (46% and 45% susceptibility, respectively). The susceptibility rates for amikacin and gentamicin also declined from 2017 to 2018. The European Antimicrobial Resistance Surveillance Network (EARS-Net) database reports that in 18 countries surveyed, more than 50% of *A. baumannii* isolates were non-susceptible to carbapenems and aminoglycosides.^35^ Recently, the National Institute for Communicable Diseases (NICD) reported on antimicrobial susceptibility patterns of ESKAPE (*Enterococcus faecium, Staphylococcus aureus, Klebsiella pneumoniae, A. baumannii, P. aeruginosa, Enterobacter* species). Organisms isolated from patients with bacteraemia in South Africa between 2016 and 2018. In this report, it is noted that for *A. baumannii* isolates from the public sector, there was a decrease in susceptibility for imipenem and meropenem between 2016 (27% and 25%, respectively) and 2017 (both 19%), remaining stable in 2018. The susceptibility rates for amikacin and gentamicin equally decreased between 2016 (44% and 32%, respectively) and 2017 (37% and 23%, respectively).^36^ Despite the overall decrease in *Acinetobacter* species over the years as described, these rates and trends are worrying and highlight the limited therapeutic options for *A. baumannii* locally and globally^37^; it is particularly concerning as *Acinetobacter* species was found to be the most prevalent NFGNB in our centre.

Contrary to the *Acinetobacter* species, there was a significant increase of *Pseudomonas* species during the study period (23% to 28%) with a slight majority isolated from the medical wards (39%), versus 36% from the surgical wards. The susceptibility rates for *Pseudomonas* species to the first line antipseudomonal drugs piperacillin-tazobactam and ceftazidime were higher than the carbapenems. Our data show a decrease in susceptibility to imipenem (75% to 71%) and meropenem (81% to 68%) over the study period. The susceptibility rates data obtained from various European countries also show susceptibility to the carbapenems below 50% among *P. aeruginosa* isolates, considerably lower still than in this study.^35^ The NICD report on the ESKAPE pathogens from the public sector showed that there were no changes in susceptibilities for this organism to imipenem and meropenem (between 76% and 78%) between the years 2016–2018.^36^ This higher resistance rate to the carbapenems compared to piperacillin-tazobactam and ceftazidime may have been driven by the widespread use of carbapenems globally.^38,39,40^

The reduced susceptibility of *Acinetobacter* and *Pseudomonas* species to carbapenems described here may be a result of overuse of these agents. Before 2018, carbapenems (imipenem or meropenem) were recommended as empiric therapy for nosocomial bloodstream infections with GNB. The current empiric antimicrobial therapy used at this hospital selected in 2019 is a carbapenem-sparing combination of amikacin and piperacillin-tazobactam. Our findings suggest that this empiric therapy was a reasonable choice for the most common NFGNB in our setting; however, further susceptibility analyses will be needed going forward to determine whether this change has had an impact.

One of the challenges regarding *S. maltophilia* is its intrinsic antimicrobial resistance to a variety of antimicrobials, especially carbapenems. The overuse of carbapenems is thought to be a driver for selection of *S. maltophilia* in patients who are heavily immunosuppressed (e.g., neutropenic).^31^ Although the numbers are minimal, this may explain why this study showed that more *S. maltophilia* isolates were isolated from the medical wards (*n* = 29; 39.7%) as these wards include the haematology patients. We were, however, unable to separate out the haematology patients to examine this further as they are often mixed with the non-haematology patients in the ward. The susceptibility to TMP-SMX was only 75% in our analysis. However, it has been shown that there is in vitro synergistic activity in combination with other antimicrobial agents even if TMP-SMX shows resistance.^34^ At this hospital, combination therapy for TMP-SMX non-susceptible strains is therefore recommended. Antimicrobial susceptibility testing for *S. maltophilia* poses a challenge as the results are particularly affected by factors such as the method of testing, culture medium, incubation temperature and even the interpretation of results.^41^ Clinical and Laboratory Standards Institute only provides minimum inhibitory concentration (MIC) breakpoints and not zone diameter breakpoints for ceftazidime and MICs were not available for all the isolates.

The study showed a high incidence of XDR *Acinetobacter* (60%) and *Pseudomonas* species (14%). Colistin is sometimes the only option remaining for treatment of infections with these organisms. Due to the increased use of colistin, there has been an emergence of resistance to this agent.^42^ Colistin has no activity against *B. cepacia* and some strains of *S. maltophilia*^43^ and the slight increase in the number of these isolates shown in this study, albeit not significant, might be due to the increase in the use of colistin to treat infections caused by XDR organisms.^44^ Additionally, an increase in the number of *B. cepacia* isolates is considered a possible indication of overuse of broad-spectrum antibiotics.^45^

There are several limitations to our study. It was a single centre study. Due to the retrospective nature of the analysis, we were unable to confirm any of the laboratory results. The lack of colistin MIC data. As only data from the laboratory database was included, the clinical significance of these isolates could not be ascertained. Even though only isolates from sterile sites were included, it remains challenging to determine whether the isolates were true pathogens or contaminants, particularly because NFGNB are common colonisers in the hospital environment. Occasionally, due to antibiotic disk stock shortages, AST for certain antibiotics was not available and antibiotics that have not been tested cannot be used for patient management. An alternative antibiotic that is susceptible would therefore be recommended. Clinical syndromes associated with NFGNB as well as specific risk factors for these infections were not established. The AST patterns for *B. cepacia* were analysed despite the CLSI recommendations that organisms with isolates of less than 30 are excluded, and the results should be interpreted with caution. There was a lack of denominator data to report prevalence.

The CLSI and the European Committee on Antimicrobial Susceptibility Testing (EUCAST) recommend using manual broth microdilution (BMD) for colistin susceptibility testing,^31^ which was conducted at a referral laboratory during the study period. Colistin susceptibility testing by BMD was not performed for the majority of our isolates and was therefore not included. There was no differentiation possible between MDR/XDR *Pseudomonas* species because of the limitation in the number of antimicrobial classes tested. We also did not have MICs for *S. maltophilia* and ceftazidime as recommended by the CLSI, so we did not include this in the analysis. A small number of the NFGNB failed to be identified by routine methods available on site (*n* = 33) and were released as NFGNB. Additional identification methods such as Matrix-assisted laser desorption/ionisation-time of flight (MALDI-TOF) mass spectrometer (MS) may have assisted in these cases.^46^ Lastly, NFGNB other than the four main species detailed in this analysis were grouped together and included when analysing the AST patterns based on disk diffusion and automated susceptibility results; CLSI does not recommend this, and these results should therefore also be interpreted with caution.

## Conclusion

The study showed a considerable burden of MDR and XDR *Acinetobacter* and *Pseudomonas* species and an emergence of NFGNB with intrinsic multidrug resistance (*S. maltophilia* and *B. cepacia*). The choice of empiric antibiotics remains a challenge due to the variability of organism. The current AST patterns show that the use of piperacillin-tazobactam and amikacin as empiric therapy for nosocomial infections was appropriate during the study period. However, ongoing surveillance is necessary to monitor and support antimicrobial stewardship practices. Improved antibiotic stewardship and infection control practices are needed to ensure rational antibiotic prescribing and slow down the emergence and spread of MDR NFGNB in our healthcare setting.

## Appendix 1

**TABLE 1-A1:** Other non-fermenting Gram-negative bacilli.

Organism	*n*	%
NFGNB (no speciation)	33	49.2
*Alcaligenes* species	7	10.4
*Aeromonas hydrophila*	5	7.5
*Brevundimonas diminuta/vesicularis*	4	6.0
*Achromobacter xylosoxidans*	3	4.5
*Chryseobacterium indologenes*	3	4.5
*Methylobacterium mesophilicum*	3	4.5
*Brevundimonas diminuta*	2	3.0
*Oligella urethralis*	2	3.0
*Psychrobacter phenylpyruvicus*	2	3.0
*Ralstonia pickettii*	2	3.0
*Ochrobactrum anthropi*	1	1.5

**Total**	**67**	**100.0**

NFGNB, non-fermenting Gram-negative bacilli.
